# Motor Cost Influences Perceptual Decisions

**DOI:** 10.1371/journal.pone.0144841

**Published:** 2015-12-16

**Authors:** Encarni Marcos, Ignasi Cos, Benoît Girard, Paul F. M. J. Verschure

**Affiliations:** 1 Laboratory of Synthetic Perceptive, Emotive and Cognitive Systems (SPECS), Universitat Pompeu Fabra, Roc Boronat 138, 08018, Barcelona, Spain; 2 UPMC – Sorbonne Universites, Univ Paris 06, UMR7222, ISIR, 4 Place Jussieu, 75005, Paris, France; 3 CNRS-UMR 7222, ISIR, 4 Place Jussieu, 75005, Paris, France; 4 Institució Catalana de Recercai Estudis Avançats (ICREA), Passeig Lluís Companys 23, 08010, Barcelona, Spain; The University of Western Ontario, CANADA

## Abstract

Perceptual decision making has been widely studied using tasks in which subjects are asked to discriminate a visual stimulus and instructed to report their decision with a movement. In these studies, performance is measured by assessing the accuracy of the participants’ choices as a function of the ambiguity of the visual stimulus. Typically, the reporting movement is considered as a mere means of reporting the decision with no influence on the decision-making process. However, recent studies have shown that even subtle differences of biomechanical costs between movements may influence how we select between them. Here we investigated whether this purely motor cost could also influence decisions in a perceptual discrimination task in detriment of accuracy. In other words, are perceptual decisions only dependent on the visual stimulus and entirely orthogonal to motor costs? Here we show the results of a psychophysical experiment in which human subjects were presented with a random dot motion discrimination task and asked to report the perceived motion direction using movements of different biomechanical cost. We found that the pattern of decisions exhibited a significant bias towards the movement of lower cost, even when this bias reduced performance accuracy. This strongly suggests that motor costs influence decision making in visual discrimination tasks for which its contribution is neither instructed nor beneficial.

## Introduction

Perceptual decision making has been mainly studied by using tasks in which humans or animals are required to discriminate a visual property of a stimulus [1, 2, 3]. It has been shown that, for example, when subjects discriminate the direction of coherent motion in a random-dot motion (RDM) task neural activity builds up at a rate related to stimulus strength and that, consequently, reaction time shortens and accuracy improves as the coherence of motion is increased (Ibid). In most of these tasks decisions are reported through oculomotor responses and therefore they have provided a very limited opportunity to study any possible influence of motor factors on decision making. However, whether in the lab or during natural behavior, decisions are typically reported by performing a motor action that generally have a variety of costs and that therefore might exert an influence on the visual assessment. Indeed, recent studies have shown that the motor system is exquisitely sensitive to subtle differences in biomechanical costs which influence not only movement implementation [4], but also decisions between them [5, 6]. The question we explore here is whether this influence also extends to perceptual discrimination tasks or, by contrast, perceptual decisions are only dependent on the visual stimulus and perfectly orthogonal to motor costs.

A growing amount of evidence is revealing that factors others than perceptual ones, such as specific asymmetries in the expected payoff associated to a given choice or in the prior probability that a given choice is correct, have an influence on perceptual decisions [7, 8, 9]. These effects are all consistent with the notion that during perceptual decision-making the brain employs a strategy aimed at maximizing the expected value of the choices it makes and minimizing costs [1]. Here, we investigate whether the consideration of motor costs, in this case the biomechanical cost of the reporting actions, could possibly spill into the assessment of visual stimuli even when they are orthogonal to task performance. In fact, if motor costs were influential onto visual decisions, this would imply that the motor system is intrinsically implicated onto perceptual decision making [10], in spite of the reduction of task performance. To test this, we presented subjects with a modified version of the classic RDM task [11] in which the costs of the reporting actions implied the opposite biomechanical cost, consistently with a well-established paradigm for motor decision making [5, 6]. We varied both the perceptual evidence (noise) of individual trials as well as the costs of the two actions used to report the choice. As a control we allowed subjects the option to “give-up” when they did not feel confident enough to make an informed guess by selecting a third target which demanded the lowest motor cost of all. Subjects were allowed to make their choice at any time, but each trial had a fixed duration to discourage random guessing.

Importantly, our task was explicitly defined as a purely perceptual discrimination task. That is, subjects were instructed to detect the direction of motion in a visual display and the movement was merely a means to report that decision. Therefore, if in any given trial a subject perceived motion to the right and then made a leftward movement then they were taking motor costs into account when reporting perceptual decisions. As to control for the possibility that subjects might by default select the less effortful of both choices on difficult trials, we incorporated a third “give-up” option, which implied an even lesser cost than the least of both previous options, which subjects were instructed to select whenever they did not feel confident enough about the visual stimulus. Under these conditions, if subjects exhibited a bias in their choices, it would mean that rather than making decisions based on visual information only, as dictated by the task instruction, they were also allowing costs relative to the movement, but orthogonal to the task, to interfere with the decision-making process.

## Materials and Methods

### Experimental design

#### Participants

**Fig 1:**
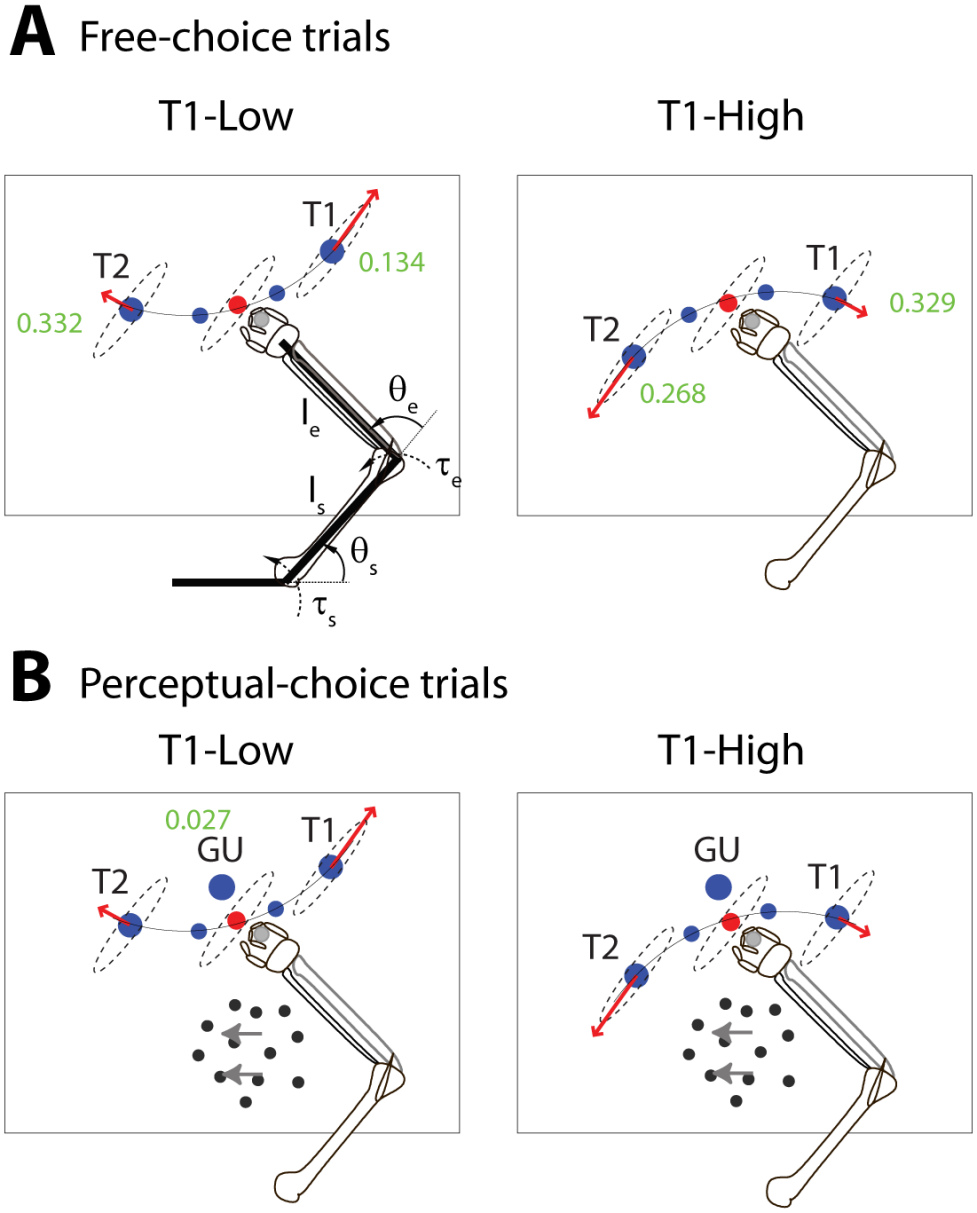
Experimental setup. **(A)** Free-choice trials. The two possible configurations of targets: *Left*, T1-Low configuration; *Right*, T1-High configuration. The thin curved lines indicate the average trajectory to perform when reporting a selection of target T1 or target T2. Starting from the red central circle the movement should pass over the via-point (small blue circle) to reach T1 or T2. The ellipse centered at the red circle (not shown to subjects) indicates the arm’s mobility ellipse. In the T1-Low configuration the final part of the movement trajectory to T1 is aligned with the major axis of the ellipse, whereas the arrival trajectory to T2 is aligned with its minor axis (read arrows). The opposite occurs in the T1-High configuration. **(B)** Perceptual-choice trials. Subjects were required to detect the net motion coherence of the moving dots that appear on the screen and to report it reaching T1 (right motion) or T2 (left motion) in one of the two possible configurations: T1-Low or T1-High. An additional third target (“give-up”-GU) was placed in the same central position in both configurations. Numbers next to the targets indicate the total estimated the muscle work required to reach that target (in Joules; [5]). A model of the right arm used to calculate the biomechanical cost of actions is shown in Left panel in (A). We modeled the arm as a two-segment rigid body rotating around the shoulder and elbow joints. *θ*_*s*_ and *θ*_*e*_ are the shoulder and elbow angles, *l*_s_ and *l*_e_ are the upper arm and forearm lengths, and *τ*_s_ and *τ*_e_ are the shoulder and elbow torques, respectively.

**Fig 2:**
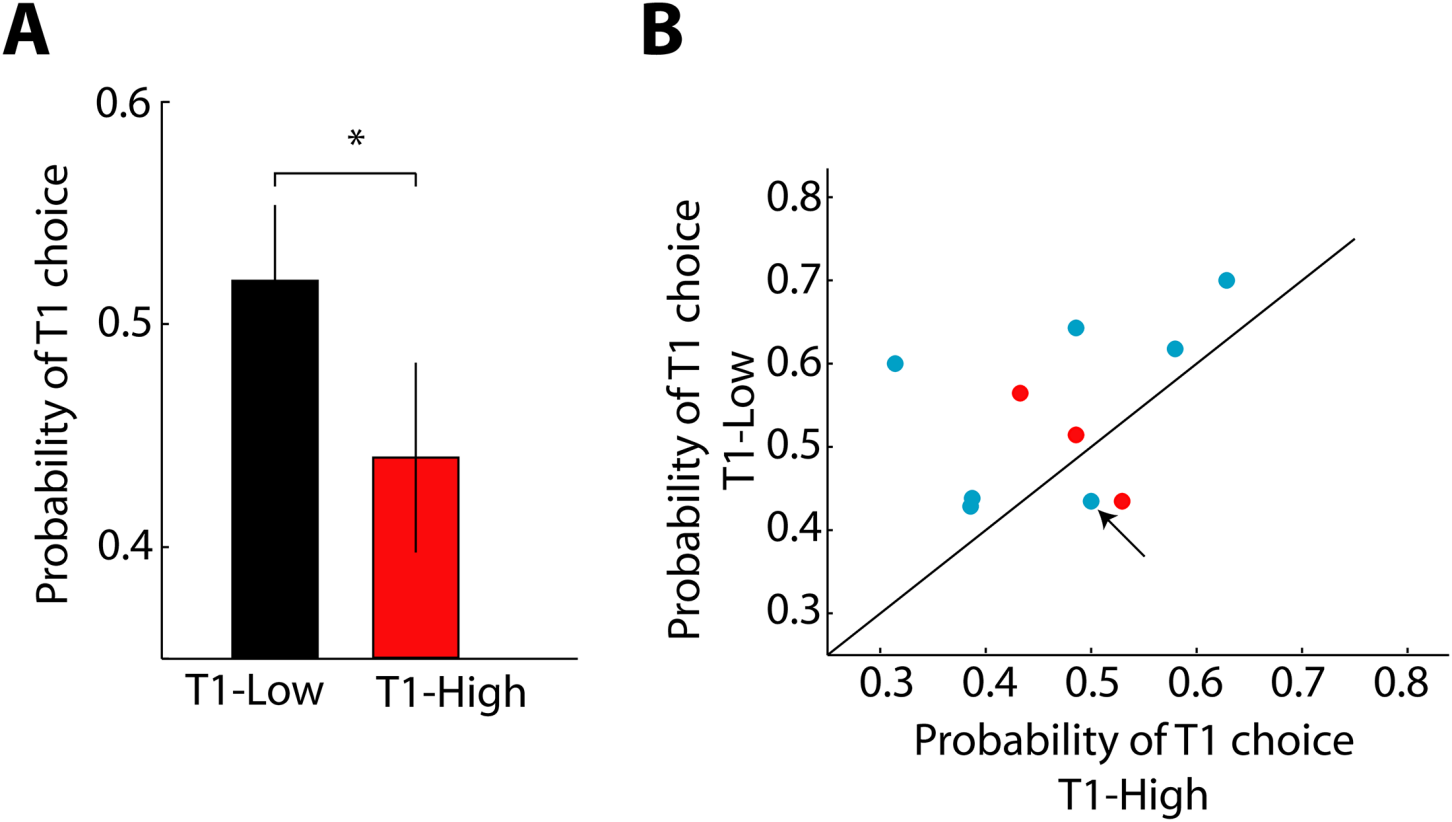
Behavioral performance during free-choice trials. **(A)** Probability of selecting T1 for the T1-Low (black) and T1-High (red) configurations (n = 11; paired-samples t-test, *t = 2*.*277*, * p < 0.*05*). Error bars show SEM. **(B)** Comparison of the probability of selecting T1 in T1-High (x-axis) and T1-Low (y-axis) for individual subjects. Consistent with (A), the majority of the dots are above the diagonal (nine out of eleven subjects). Red dots correspond to the ones that performed solely free-choice trials (n = 11) whereas cyan dots correspond to the subjects that performed both free-choice and perceptual choice trials (n = 8). The black arrow indicates a subject that was excluded from the perceptual bias analyses due to the individual lack of sensitivity to action cost.

**Fig 3:**
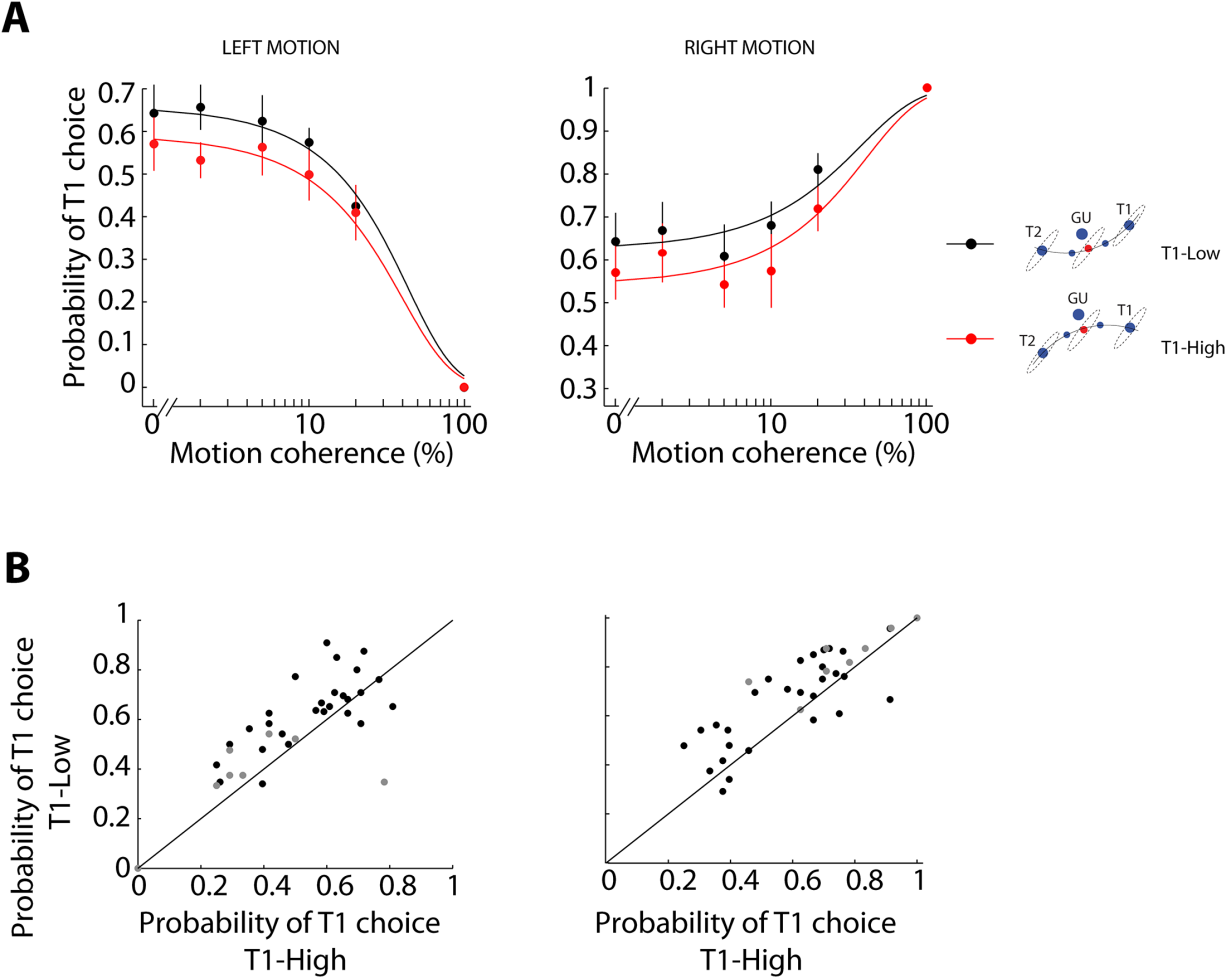
Behavioral performance during perceptual-choice trials. **(A)** Mean probability of T1 choice across all subjects for right and left motion for different values of motion coherence for T1-Low (black) and T1-High (red) configurations (n = 7; Repeated measures ANOVA showed a main effect for coherence and for targets configuration: *F(10*,*60) = 54*.*53*, *p<0*.*001* and *F(1*,*6) = 7*.*91*, *p<0*.*05*, respectively). Solid lines are fits to the data with a psychometric function (*R = 0*.*99* for T1-Low and *R = 0*.*98* for T1-High, *p < 0*.*001* in both cases; one-sample Wilcoxon signed-rank test, *p<0*.*05*; see Materials and Methods). Error bars are SEM. **(B)** Individual subjects probability of selecting T1 for left (Left panel) and right (Right panel) for a specific level of motion coherence, solid black circles for 0%, 2%, 5% and 10% amount of motion coherence and solid gray circles for 20% and 100%. Consistent with (A), the majority of the dots are above the diagonal.

Eleven right-handed subjects (7 females, aged 24–34 years) with normal or corrected-to-normal vision performed the free-choice trials of the experiment (Fig 1A) and eight of these eleven subjects (5 females, aged 24–34 years) also performed two more sessions that contained free-choice trials and perceptual-choice trials (Fig 1B). One of these eight subjects did not show an effect of action cost in the free-choice trials (Fig 2B) and therefore his/her data was not considered in the analyses of Fig 3. All participants were naive to the purpose of the experiment and gave informed written consent before it started. The experiment was approved by the local Ethics Committee for Clinic Research Ethics (CEIC) at the Parc de Salut MAR in Barcelona.

#### Experimental task

Human participants performed two kinds of trials: free-choice trials (Fig 1A) and perceptual-choice trials (Fig 1B). In the first case, subjects were asked to freely choose between two targets: target 1 (T1) and target 2 (T2). In the second case, subjects were instructed to detect the direction of motion of a number of dots presented in a visual display and to report their decision by making a movement towards T1 or T2. In the case they could not reliable detect the direction of motion, subjects were instructed to select a third “give-up” option. The two main targets (T1 and T2) were presented in two possible configurations: T1-Low or T1-High (Fig 1), where “Low” and “High” denote the relative biomechanical cost of the movements from the central origin towards T1 or T2. Importantly, the launching costs of movements towards either target were equalized by ad-hoc via-points which guided each movement’s initial phase. As such, the differences in biomechanical cost between movements were due only to each target’s arrival phase.

Each individual trial began when the central cue, or origin cue, (red central dot, Radius 0.47cm) was shown on the screen. Subject had to move the stylus into the origin cue and held it there for 500ms so that the stimuli defining the potential trajectories were shown. Each potential trajectory was defined by the central cue, a via-point (cyan dot, Radius 0.55cm) and a target (cyan dot, Radius 0.85cm). In the free-choice trials subjects were asked to freely choose one of the two targets by performing a horizontal trajectory. In the perceptual-choice trials, a visual stimulus consisting of dots moving towards left or right, with a net motion coherence of 0%, 2%, 5%, 10%, 20% or 100%, appeared 200ms after the presentation of the targets. In this case, subjects were required to detect the direction of the moving dots and to report it as fast as possible by selecting either target T1 or T2 or to select a "give-up" option if they were not able to detect the dots direction of movement. The via-point and target cues changed to a lighter cyan as the stylus moved over them.

The dynamic random dots that were displayed in a 5° circular aperture with a dot density of 16,7 dots per deg^2^ per s [11, 12, 13]. Dots were placed in a random position or in a subsequent right or left position every three frames (50ms). Coherently moving dots were displaced to produce 6° s^-1^ motion.

The trials were divided into three sessions. The first session contained only free-choice trials and was used to characterize the influence of action cost on the subjects' decision in the absence of any other sensory information. It contained two types of trials: two-target trials (N = 140) which were used to assess subjects' choices and one-target trials (N = 120) which were used to ensure that subjects had substantial experience performing all of the movements. The trials were divided into three blocks of 100, 80 and 80 trials, respectively, in which 40 of them were one-target trials and all conditions were balanced (same number of trials) within a block. The two remaining sessions consisted of both free-choice trials and perceptual-choice trials. These two sessions were divided into four blocks of trials: the first block contained free-choice trials (40 trials with 24 two-target trials and 16 one-target trials) whereas the other three blocks had exclusively perceptual-choice trials (96 trials with interleaved T1-Low and T1-High configurations). In each experimental session, the first block was used as an exploratory task to allow subjects to experience the action cost of reaching each target. In the perceptual-choice trials, when in a given trial subjects selected the "give-up" option a new trial with the same motion coherence was generated and placed at the end of the block’s sequence. With this procedure we ensured that we had enough trials with responses that reflected the detection of motion coherence. Subjects were unaware of the total number of trials of each block and that trials with give-up choices were placed at the end of the sequence. To avoid that the duration of the session was dramatically increased by this procedure we limited the number of trials in each block to 120 trials. A trial had a fixed total duration of 4s, which means that if subjects selected a target before the end of the trial the screen appeared white for the remaining time. This was used to discourage random guessing. The inter-trial interval had a duration of 500ms. All subjects experienced the same pseudorandom sequence of trials that differed between blocks. No feedback was given to subjects at the end of a trial to avoid any additional influence that this factor might have in their behavior.

RTs were calculated as the difference between motion onset and the time in which the cursor of the digital pen left the central cue.

The experimental setup consisted of a touchscreen computer (Sony VAIO L Series Touchscreen AIO PC 24'', resolution of 1920x1080). Subjects sat on a chair facing the horizontal screen at approx. 20cm, and were instructed to maintain that position throughout the experiment. They were asked to maintain their right arm on the horizontal plane parallel and above the horizontal screen while grasping a digitizing stylus that gently leaned on the touchscreen. Subjects performed planar movements by sliding the stylus on the touchscreen, while maintaining it vertical. The position of the stylus’ tip was sampled at 60Hz by the screen software and was recorded by our ad-hoc built task control software. Subjects were visually controlled by the experimenter to ensure that these requirements were fulfill during the experiment.

### Characterization of motor (biomechanical) cost

Previous studies determined that the biomechanical costs associated to movements along different directions could be predicted in anticipation of movement onset and biased the selection between movements [5, 6]. The biomechanical cost of actions was characterized by the degree of alignment of the end-point trajectory of movement with the major (low cost) or minor (large cost) axis of the ellipse of mobility. The transformation from the arm inertia tensor into end-point mobility one is calculated as:

$$W\left(\theta \right)=J\left(\theta \right)I^{-1}\left(\theta \right)J\prime \left(\theta \right)$$ (1)

where *W(θ)* is the inverse of the inertia tensor *I(θ)* and *J(θ)* is the Jacobian of the arm that transforms the mobility tensor from joint space into end-point space. Using a planar, simplified model of the arm consisting in two rigid body segments, the resulting formulation of the inertia tensor can be expressed as:

$$I\left(\theta \right)=\left[\begin{array}{cc} m_{s}c_{s}^{2}l_{s}^{2}+m_{e}l_{s}^{1}+m_{e}c_{e}^{2}l_{e}^{2}+2m_{e}c_{e}l_{s}l_{e}cos\left(\theta_{e}\right) & m_{e}c_{e}l_{s}l_{e}cos\left(\theta_{e}\right)+m_{e}c_{e}{}^{2}l_{e}^{2}\\ m_{e}c_{e}l_{s}l_{e}cos\left(\theta_{e}\right)+c_{e}^{2}l_{e}^{2} & m_{e}c_{e}^{2}l_{e}^{2} \end{array}\right]$$ (2)

where *θ*_*s*_ and *θ*_*e*_ are the shoulder and elbow angles as defined in Fig 1, m_s_ and m_e_ are the averaged mass and c_s_ and c_e_ are the averaged mass center distances. For our calculations we used: m_s_ = 1.76kg, m_e_ = 1.65kg; c_s_ = 0.475, c_e_ = 0.42 [14]. Using the inertia tensor we can derive the muscle torques (*τ*_*m*_) predicted for the subjects’ trajectories (see [5] for a detailed account of this calculation) and consequently the muscle work (MW) necessary to move the arm from the origin to the target:

$$MW=\int \begin{array}{l} \theta_{\text{target}}\\ \theta_{origin} \end{array}\tau_{m}d\theta$$ (3)

Where *θ* are the planar arm angles expressed in radians. The resulting estimates of muscle work for each movement and configuration are calculated post-hoc and shown above the targets of Fig 1.

In essence, the axes of the mobility ellipse match the directions of movement implying maximum and minimum muscle work. Hence, to define two configurations with opposite biomechanical cost, we designed a T1-Low and aT1-High configuration. In the T1-Low configuration, the trajectories arriving to T1 and T2 were aligned with the major and minor axis of the ellipse of mobility, respectively. The alignments were reversed in the T1-High configuration (see Fig 1).

### Data analyses

To assess the bias in the probability of T1 choices for right/left motion direction between T1-Low and T1-High during the perceptual-choice trials we performed a logistic regression. Therefore, we fitted the behavioral performance relative to the motion direction with a logistic psychometric function [7]:

$$P_{T1}=\frac{1}{1+e^{-(\beta_{0}+\beta_{1}c^{\prime }+\beta_{2}I)}}$$

where *β* coefficients are free parameters, *c’* is the motion coherence level and *I* has a value of 1 when the targets are arranged in the T1-High configuration and 0 when they are placed in the T1-Low configuration. Although plotted separately, the fitting of the curves was done considering left and right motion direction together. To investigate the bias in performance due to the configuration of the targets, we evaluated the null hypothesis that the arrangement of the targets does not bias choices made by the subject in a way related to the cost of actions (this occurs when the related parameter, *β*_2_, has a value greater or equal to 0, *β*_*2*_ ≥ 0). The significance of the results was obtained by comparing the distribution of the individual *β*_*2*_ parameters with the null hypothesis (H_0_: *β*_*2*_ ≥ 0; one-tailed one-sample t-test) and by using a repeated measures ANOVA test (with coherence and targets configuration as factors). We estimated the strength of the effect in terms of motion coherence with the ratio *β*_*2*_ */β*_*1*._

Reaction time curves were fitted by a hyperbolic tangent function:

$$RT\;=\;\frac{\beta_{3}}{\beta_{4}\;\cdot \;c\prime }\text{ tanh}\left(\beta_{3}\;\cdot \;\beta_{4}\;\cdot \;c^{\prime }\right)\;+\;t_{R}$$

where *β*_3_ and *β*_4_ and *t*_*R*_ are free parameters.

## Results

To quantify the effect of motor cost in the selection of movement, we first assessed the subjects’ preference for either of the two targets in free-choice trials (Fig 1A). As expected, in the absence of moving dots, the participants exhibited a tendency to select the Low (cost) target more often than the High one (mean probability of T1 choice T1-Low: 0.52; T1-High: 0.44; paired-samples t-test, *p < 0*.*05*, Fig 2). This bias is consistent with that reported in earlier studies [5, 6]. In addition, the similarity between the movement velocity profiles for each target selection and configuration (S1 Fig) rules out the possibility of speed as responsible for target preferences and confirms the major influence of biomechanical factors on the selection of motor actions.

Second, we investigated whether the different biomechanical cost of the reporting actions influenced visual decisions during a RDM task (perceptual-choice trials). In this case, rather than making a free choice, the participants were instructed to report the perceived direction of motion of the dots by reaching towards either T1 or T2. The geometrical arrangement of T1 and T2 could be presented either in its T1-Low (T1 small cost, T2 large cost) or T1-High configuration (T1 large cost, T2 small cost), see Fig 1B. We observed that the difference in the selection of T1 and T2 between the T1-Low versus T1-High configurations is also present when subjects report the direction of motion in perceptual-choice trials: the probability of T1 choices for left- and rightward motion depended on the biomechanical cost of the action required to reach the targets (Fig 3A). Subjects selected T1 significantly more often for both directions of motion when the targets were in the T1-Low configuration as compared to T1-High (Repeated measures ANOVA with T1 choices as the variable and targets configuration and motion coherence the as factors, *F(1*,*6) = 7*.*91*, *p<0*.*05*). This suggests that subjects have a tendency to detect rightward motion more frequently when the targets are placed on the T1-Low configuration than when the targets follow the T1-High configuration. Indeed, this effect can only be explained by fitting the probability of selecting T1 with a psychometric function that comprises three free parameters: an initial perceptual bias, the level of motion coherence and the configuration of the targets. The third parameter (*β*_*2*_) is fundamental to account for the differences on T1 choices between the two targets configurations (*β*_*2*_ = *-0*.*34 ± 0*.*14* SEM; one-tailed one-sample t-test, *p<0*.*05*, see Materials and Methods). The correlation coefficients between the fitting of the curves (psychometric functions) and the real data points are greater than 0.85 (*R>0*.*85*) and significant (*p<0*.*001*) for all individual subjects. The effect of the cost of actions is equivalent to an additional ~6.6% of rightward motion added to the stimulus in T1-Low with respect to T1-High. Fig 3B shows a consistent effect across individual subjects. An analysis of mean RTs of subjects revealed no significant differences in response times between conditions (see Material and Methods): mean RT of 1.18s (±0.09s SEM) and 1.25s (±0.13s SEM) for right motion and T1-Low and T1-High, respectively, and 1.37s (±0.15s SEM) and 1.46s (±0.11s SEM) for left motion and T1-Low and T1-High, respectively. Similar to the bias reported by Resulaj et al. [15], the choices observed exhibit a mild lateral bias towards T1 in both T1-Low and T1-High (for 0% of motion coherence the probability of selecting T1 is 0.64 for T1-Low and 0.57 for T1-High). To control for the potential additional biases unrelated to biomechanics, we performed a control experiment in which subjects used a keyboard to report the motion direction of the moving dots. The proportion of right choices was above 0.5 for all motion coherences (S2 Fig), showing the same mild lateral bias and indicating that the bias is not associated with the addition of the biomechanical manipulation.

**Fig 4:**
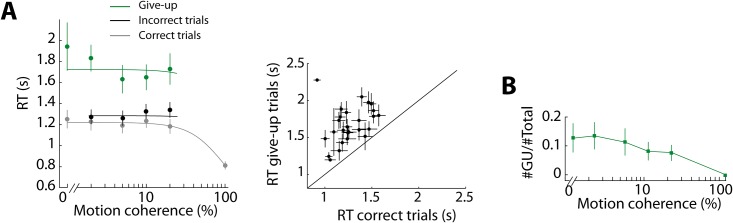
Give-up choices. **(A)** *Left panel*, mean RT for different levels of motion coherence for correct, incorrect and give-up trials (n = 8). RT was significantly longer when subjects selected the give-up target than when they correctly detected the direction of motion and selected either T1 or T2 (Repeated measures ANOVA showed a main effect for targets choice, i.e. T1/T2 or give-up, *F(1*,*4) = 40*.*51*, *p < 0*.*01*). Experimental data (dots) were fitted with a hyperbolic tangent function (see Materials and Methods). *Right panel*, mean RTs for correct versus give-up trials. Each data point corresponds to one subject and one level of motion coherence (removing one outlier data point, *RT > 3s*). Error bars are SEM. **(B)** Give-up (GU) choices for different levels of motion coherence (n = 8).

Is it possible that the influence of the biomechanical bias during perceptual-choice trials is due to a strategy of simply choosing the easier movement by default during trials in which the subject is unsure of the direction of motion? The "give-up" target was introduced into the experimental setup to specifically prevent this confound. That is, if a subject was unable to detect motion in a particular trial, they were instructed to select the “give-up” option, whose biomechanical cost was lowest of all, and move on to the next trial. As expected, “give-up” choices tended to be made with long reaction times (RTs; Fig 4A) and decreased in frequency as a function of motion coherence (Fig 4B). In addition, the proportion of "give-up" choices for motion direction depended on the targets configuration (proportion of "give-up" choices for right motion: 0.168, SD = 0.777, for T1-Low and 0.146, SD = 0.771, for T1-High; n = 8, paired-samples t-test, *t = 2*.*798*, *p<0*.*05*). Subjects selected more often the give-up choice when the dots favored the direction reported by the less costly action. This indicates that, in these cases, rather than incorrectly detecting the direction of motion subjects give up. Altogether, these results confirm that when subjects did choose T1 or T2, they presumably acted on the basis of a non-negligible amount of certainty on the motion coherence rather than opting for the easiest action, confirming that action costs bias perceptual decision.

## Discussion

Here, we have investigated the influence of motor cost in a perceptual discrimination task. To that end, we have modified a RDM task by varying the biomechanical cost of the reporting actions and by adding a "give-up" option. Our results show that even the subtle differences of motor cost between T1 and T2 movements can significantly bias the participants' decisions towards the least costly option, although this was not part of the task instruction and was detrimental to the participants performance. We have quantified the bias in the perceptual decisions as comparable to an additional amount of motion coherence of about 6.6% favoring the direction that required a movement with lower motor cost (virtual increase of motion towards right in the T1-Low case compared to the T1-High case).

To control that subjects did not simply choose the action requiring the lesser cost when they were uncertain of the direction of the moving dots we introduced a third, "give-up", option. We instructed the participants to select it whenever they were uncertain about the direction of the moving dots. The presence of this option suggests that T1 or T2 selections occurred only when the subjects had detected rightward or leftward motion, respectively, as indicated by the decrease of give-up choices with an increase in the amount of motion coherence [16]. The significant difference in the proportion of give-up choices for right motion in T1-Low (0.168) compared to T1-High (0.146) shows that subjects often prefer to give up rather than selecting the incorrect target when this requires the action with the highest cost. Altogether, these results suggest that subjects selected T1 or T2 on the basis of some direction of motion detected towards right or left, respectively.

We interpret the perceptual bias observed in the participants' decisions as a consequence of an asymmetric motor cost related to each option, suggesting that stronger perceptual evidence is needed to select the most costly option. These results are consistent with previous theoretical work [17], in which a synergy between perception and behavior was reported. Given the experimental data that we have presented here, we can claim that motor cost influences perceptual decisions even when it is orthogonal to the task instruction and it compromises performance. However, further research is required to identify the specifics of this influence, whether the motor cost causes a perceptual artifact, whether it uniquely influences the competition between reporting actions or whether it is a constant that influences the entire process as suggested by a recent study [18].

The main result of the biomechanical influence on perceptual decision making also indicates that choices exhibit a mild lateral bias towards T1 in both T1-Low and T1-High conditions, in a similar fashion to the bias reported by Resulaj et al. [15]. As a control for this, we performed an additional experiment in which subjects used the keyboard to report the motion direction of the moving dots and we observed the same mild lateral bias in this case. Therefore, in our case, this bias may not be attributed to any conflict between motor and perceptual costs, but the real factor causing it remains unexplained. One explanation to it might be that subjects were right-handed and this might influence their perception of motion direction causing a bias towards right or, simply, that, for some reason, they prefer that direction of action movement. If any of these possibilities would be the case, left-handed subjects would show the opposite effect. Further experiments following this line of research might help to clarify the possible causes of the lateral bias.

Many studies of perceptual decisions have shown that the timing and accuracy of choices are well predicted by a model in which neural activity builds up to a decision threshold at a rate related to stimulus strength [1, 2,3]. Following the dynamics of those models, the motor cost influence on perceptual decisions could be explained by a modulation of either the decision threshold, the building up rate or the baseline from which the process starts. However, if this would be the case, contrary to what we have observed, RTs in each condition should significantly differ, leading to faster RTs for low motor cost cases than for high cost ones. The lack of this effect in our results might be due to the presence of the "give-up" target that might obscure it. Further research without the possibility to select this option would be needed to shed light on this issue.

The influence of motor cost on perceptual decision making has been previously examined in a recent study [9] which also used a perceptual task based on target-directed reaching movements to report left or rightward motion of a random dot stimulus. In contrast to the current study, this previous study examined the influence of motor cost on change of mind [15]. The authors reported that the proportion of changes of mind was sensitive to motor cost. The current study extends and complements this result by showing that motor cost also influences the initial decision.

In our task, the presentation of the targets always preceded the onset of the perceptual stimulus. According to decision-making models with attractor dynamics [19] any information available early in the decision-making process may have a greater influence and therefore, the early presentation of motor targets might have enhanced the effect of motor cost on perceptual decisions. Additional experiments manipulating the temporal order of the presentation of motor and perceptual evidence could be conducted to investigate this matter.

Neurons in parietal and frontal areas have been shown to be involved in two-choice perceptual and motor decision-making tasks and the mean firing rate of neurons in these areas is correlated with the formation of decisions [1, 20, 21, 22]. Moreover, it has been reported that activity of neurons in the lateral intra-parietal cortex are at intermediate levels when a sure target ("give-up") is selected when compared to the selection of a non-sure target [16]. Current views on decision making attribute this lower level of activation to an urgency signal that forces any individual to make a decision even if a specific level of activation has not been reached [16, 23]. However, an alternative explanation could be that not only mean activity is critical during decision making but also factors other than that might play an important role. For instance, based on a previous study of ours [24], we may predict that the variance of the neural response could be critical in assessing the confidence of the decision being made and in deciding between giving-up or committing to a specific target. Further neural recordings from the areas involved in decision making is necessary to shed light on this theoretical prediction.

## Supporting Information

S1 Fig Average movement velocities in free-choice one-target trials. The velocities are aligned by the time of via-point crossing. The velocity profiles are shown for the selection of T1 or T2 in the two possible target configurations (as indicated in the legend). In general, the velocities were bell shaped, single peaked and similar in all conditions.
(TIF)

S2 Fig Behavioral performance in a control experiment excluding biomechanical cost. **(A)** Proportion of right motion detection in a control experiment. 3 of the 8 subjects from the main experiment performed a control experiment in which they were required to detect the direction of motion of the moving dots and to indicate it using the keyboard, where 'j' meant left direction, 'k' meant right direction and the 'space bar' was used as the 'give-up' option. The proportion of right motion detection was above 0.5 along all motion coherences showing a bias towards this direction. As in the main experiment, the number of times that subjects selected the 'give-up' option decreased as the difficulty of the trial also decreased: 25,14%, 23,43%, 15,73%, 18,18%, 3,37% and 0% of the trials for 0%, 2%, 5%, 10%, 20% and 100% of motion coherence respectively. **(B)** Probability of correct choices for different levels of motion coherence. Probability of being correct is around chance level for 0% coherence and reaches its maximum value of 1 for 100% coherence.
(TIF)
