# Supplementary figures and images for: Motor Cost Influences Perceptual Decisions
Source: PLoS One. 2015 Dec 16;10(12):e0144841. doi: 10.1371/journal.pone.0144841 (PMC4684499; doi:10.1371/journal.pone.0144841)

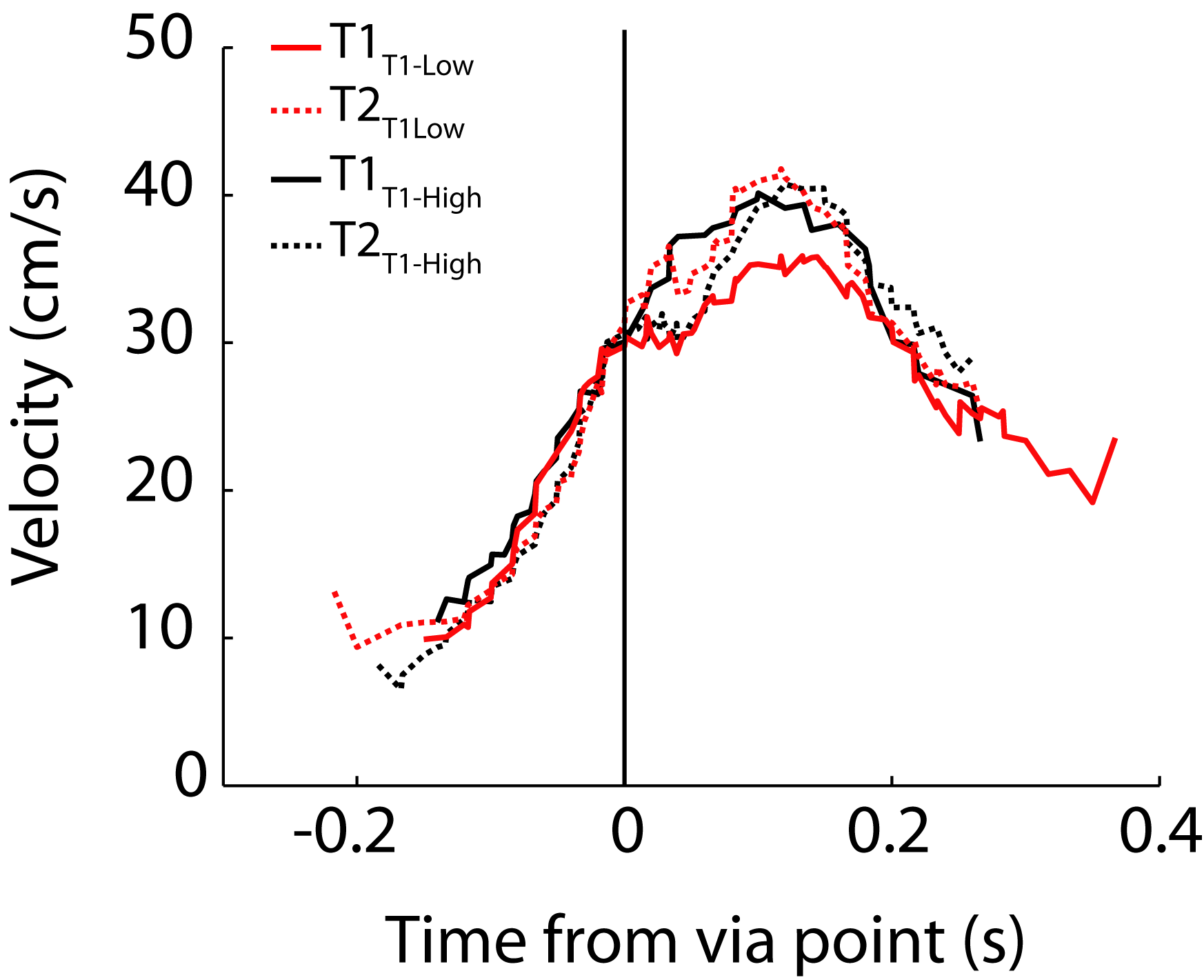

Supplement: S1 Fig — The velocities are aligned by the time of via-point crossing. The velocity profiles are shown for the selection of T1 or T2 in the two possible target configurations (as indicated in the legend). In general, the velocities were bell shaped, single peaked and similar in all conditions. (TIF) [file pone.0144841.s001.tif]

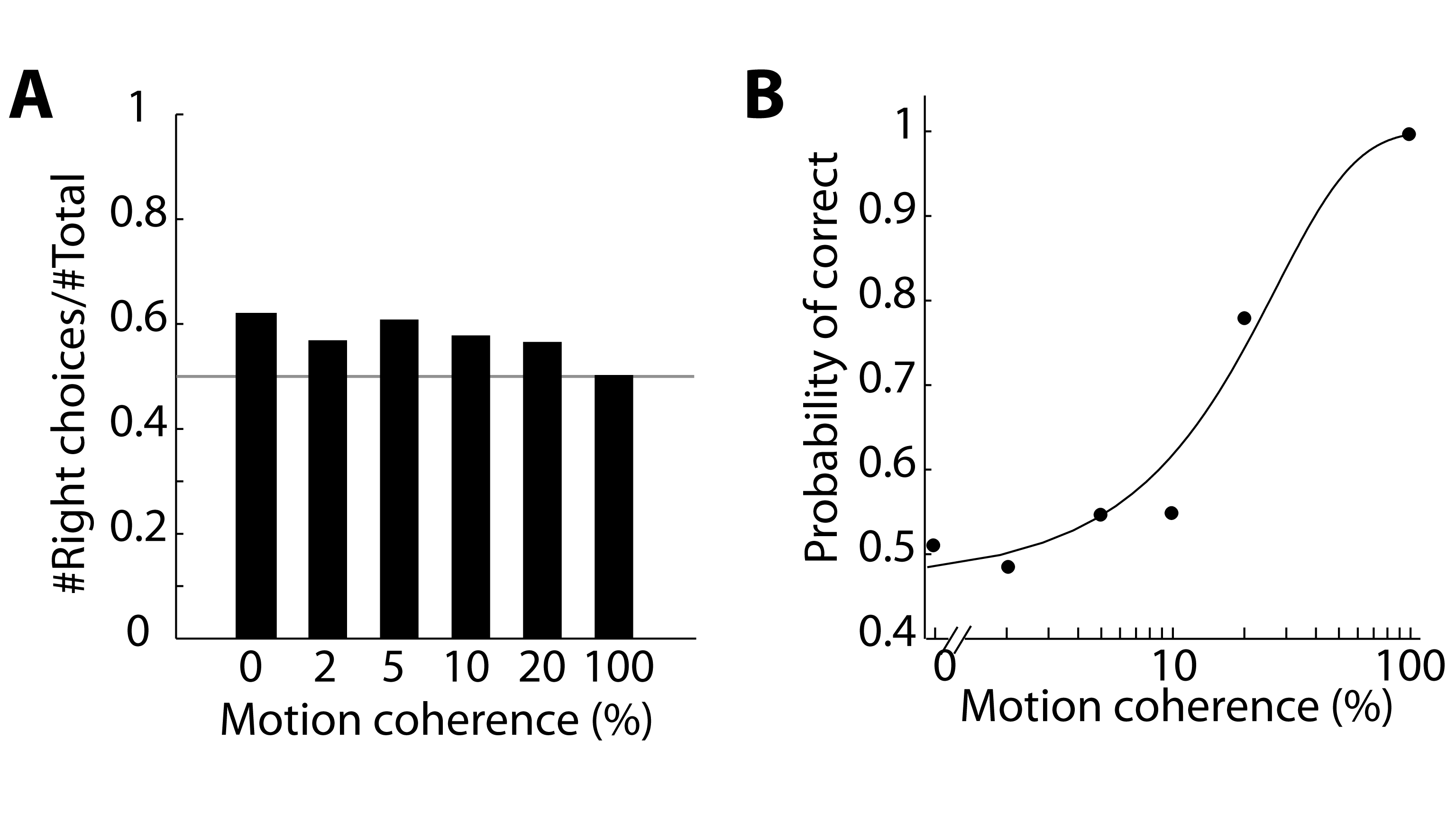

Supplement: S2 Fig — (A) Proportion of right motion detection in a control experiment. 3 of the 8 subjects from the main experiment performed a control experiment in which they were required to detect the direction of motion of the moving dots and to indicate it using the keyboard, where 'j' meant left direction, 'k' meant right direction and the 'space bar' was used as the 'give-up' option. The proportion of right motion detection was above 0.5 along all motion coherences showing a bias towards this direction. As in the main experiment, the number of times that subjects selected the 'give-up' option decreased as the difficulty of the trial also decreased: 25,14%, 23,43%, 15,73%, 18,18%, 3,37% and 0% of the trials for 0%, 2%, 5%, 10%, 20% and 100% of motion coherence respectively. (B) Probability of correct choices for different levels of motion coherence. Probability of being correct is around chance level for 0% coherence and reaches its maximum value of 1 for 100% coherence. (TIF) [file pone.0144841.s002.tif]
